# Exopolysaccharides produced by marine bacteria and their applications as glycosaminoglycan-like molecules

**DOI:** 10.3389/fchem.2014.00085

**Published:** 2014-10-08

**Authors:** Christine Delbarre-Ladrat, Corinne Sinquin, Lou Lebellenger, Agata Zykwinska, Sylvia Colliec-Jouault

**Affiliations:** EM^3^B Laboratory, Institut Français de Recherche pour l'Exploitation de la MerNantes, France

**Keywords:** marine-derived polysaccharide, biodiversity, biological activity, structure, modification, GAG-mimetic, biosynthesis

## Abstract

Although polysaccharides are ubiquitous and the most abundant renewable bio-components, their studies, covered by the glycochemistry and glycobiology fields, remain a challenge due to their high molecular diversity and complexity. Polysaccharides are industrially used in food products; human therapeutics fall into a more recent research field and pharmaceutical industry is looking for more and more molecules with enhanced activities. Glycosaminoglycans (GAGs) found in animal tissues play a critical role in cellular physiological and pathological processes as they bind many cellular components. Therefore, they present a great potential for the design and preparation of therapeutic drugs. On the other hand, microorganisms producing exopolysaccharides (EPS) are renewable resources meeting well the actual industrial demand. In particular, the diversity of marine microorganisms is still largely unexplored offering great opportunities to discover high value products such as new molecules and biocatalysts. EPS-producing bacteria from the marine environment will be reviewed with a focus on marine-derived EPS from bacteria isolated from deep-sea hydrothermal vents. Information on chemical and structural features, putative pathways of biosynthesis, novel strategies for chemical and enzymatic modifications and potentialities in the biomedical field will be provided. An integrated approach should be used to increase the basic knowledge on these compounds and their applications; new clean environmentally friendly processes for the production of carbohydrate bioactive compounds should also be proposed for a sustainable industry.

## Glycopolymers and their importance in glycobiology

Polysaccharides are natural macromolecules composed of osidic monomers and present in all organisms: microorganisms, plants and animals. Polysaccharides, such as starch, glycogen, and cellulose, are common. However, some other specific classes of polysaccharides exist based on their origin, chemical structure and function. Although polysaccharides are ubiquitous, their chemical structure varies greatly from one to the other. They have various roles within the cells, from structural involvement to numerous biological activities including interactions. Therefore, studying the polysaccharide chemical structure and structure-related activities remains a challenge for glycochemists and glycobiologists. Polysaccharides can be distinguished by their osidic composition: homopolysaccharides, which contain a single type of monosaccharide, and heteropolysaccharides composed of different osidic residues and usually displaying a regular backbone structure with a repeating unit. This repeating unit may be linear or branched and may contain up to 10 monomers as well as organic or inorganic substituents such as phosphate, sulfate, and lactic, succinic, acetic and pyruvic acids. The chemical structure including monosaccharide composition and repeating unit sequence as well as non-carbohydrate substituents is species-specific (Decho, 1990) and may vary, most of the time, with production, culture conditions and the physiological state of the organism. The linkages most commonly found between monomers are β-1,4 or β-1,3 giving a more rigid backbone vs. α-1,2 and α-1,6 for more flexible zones. The overall physical properties of polysaccharides are also influenced by the monosaccharide composition, the osidic sequence and the network formed by the single polymer chains (Poli et al., 2010). These polymers are high-molecular weight macromolecules usually above 10^6^ g mol^−1^.

Most of the polysaccharides used in the industry are extracted from plants, algae, and animals. A few of them come from bacteria and have industrial applications in the paper, food, biotechnological, environmental or health industry (Finore et al., 2014). The most famous examples of microbial used macromolecules have been listed in Table 1 (Kumar et al., 2007; Freitas et al., 2011). Owing mainly to their rheological properties, fungal exopolysaccharides (EPS) have also found several applications in the food, cosmetic and pharmaceutical industries as well as in oil recovery (Mahapatra and Banerjee, 2013). Scleroglucan produced by *Sclerotium rolfsii*, schizophyllan, a structurally similar molecule produced by *Schizophyllum commune* and pullulan from *Aureobasidium pullulans* are the most common fungal polysaccharides of high-added value (Survase et al., 2007; Mahapatra and Banerjee, 2013). Some of these polysaccharides are homolog counterparts of plant or animal macromolecules. Compared to these sources, microorganisms allow a better controlled production in bioreactors, devoid of no variation due to physiological state or season encountered for the highest organisms (Bertagnolli et al., 2014) and an easier extraction without any drastic or environmentally toxic compounds. However, downstream processing of bacterial polysaccharides still represents an important cost intensive step (Kreyenschulte et al., 2014). Moreover, microorganisms cultivation in fermenters allows the optimization of the growth and the production yield either by the study of physiology or by genetic engineering. For the high-added value pharmaceutical industry, bacterial polysaccharides can be produced at a viable economic cost. The production in controlled conditions is in agreement with the Good Manufacturing Practices (GMP) such as well-defined medium, controlled environment without the risk for viral or pathogen agents. The advantages of a bacterial source over plant, algal or animal source have made it attractive to obtain macromolecules for various industrial purposes and strengthened their study.

**Table 1:** **Examples of microbial used macromolecules (adapted from Kumar et al., 2007; Freitas et al., 2011)**.

**Exopolysaccharide**	**Source**	**Main applications**
Xanthan	*Xanthomonas campestris*	food industry as texturizing agent, petroleum industry, health care
Alginate	*Pseudomonas aeruginosa, Azotobacter vinelandii*	food hydrocolloid, wound care, drug encapsulating agent
Dextran	*Leuconostoc mesenteroides*	food industry, biomedical as plasma volume expander biotechnological supports for separation
Cellulose	*Acetobacter xylinum*	food industry, biomedical as artificial temporary skin, biotechnological separations as hollow fiber and membranes
Hyaluronic acid	*Streptococcus equi, Streptococcus zooepidemicus*	human health cosmetics
Gellan	*Sphingomonas paucimobilis*	food industry, biotechnology (culture medium gelification)
Curdlan	*Sinorhizobium meliloti, Agrobacterium radiobacter, Alcaligenes faecalis*	food and pharmaceutical industries, bioremediation
Succinoglycan	*Sinorhizobium meliloti, Alcaligenes faecalis*	food and pharmaceutical industries, oil recovery
Levans	Various	food industry (prebiotic)

Complex carbohydrates and glycoconjugates have a basic importance in biological systems and cellular processes, either physiological or pathological. They play a major role as structural agents in connective tissues, and they are ubiquitously present on cell surfaces, mediating the interaction of the cells with other cells, with the extracellular matrix, with biotic or abiotic surfaces and with other molecules. But their study that falls into the field of the glycobiology was greatly hindered by technical issues; no sequencing tool such as that existing in proteomics or genomics is available to date while glycopolymers potential chemical diversity is far greater than proteins and nucleic acids (Turnbull and Field, 2007; Merritt et al., 2013). While genes and proteins syntheses are based on a template, polysaccharide biosynthesis is regulated by a number of physiological and metabolic parameters including the availability of sugar precursors and the expression level of enzymes. The number of osidic residues combined with the anomeric configuration of the linkages and the possibility of branching result in unique complexity and diversity of carbohydrates molecules. Nonetheless, some advances in analytical techniques have revealed the vast and diverse chemical structures of carbohydrates existing in nature (Grice and Wilson, 2010); insights into the structure-function relationship allow determining their role in diverse cellular processes. Glycobiology becomes therefore a major research field allowing the understanding of human diseases and the discovery of novel therapeutic compounds.

Glycosaminoglycans (GAGs) are glycopolymers found in animal tissues and composed of uronic acid and neutral or hexosamine residues. They are covalently bound to a core protein and are the major constituent of proteoglycans. Their carbohydrate backbone is unique for each cell type. GAGs are essential for life of animals since they are involved in their development and organogenesis (De Angelis et al., 2013). Two GAG macromolecules are frequently used in various industry fields, namely hyaluronic acid and heparin.

Hyaluronic acid is a polysaccharide ranging from 500 to 1000 10^3^ g mol^−1^, with a disaccharidic linear and non-sulfated repeating unit (Table 2). It is widely used in osteoarthritis treatment as synovial fluid because of its effect on the cartilage, in ophthalmology treatments and surgery because of its visco-elastic properties, and in wound healing as well as in the cosmetic industry (moisturizing agent, wrinkle filler).

**Table 2:**
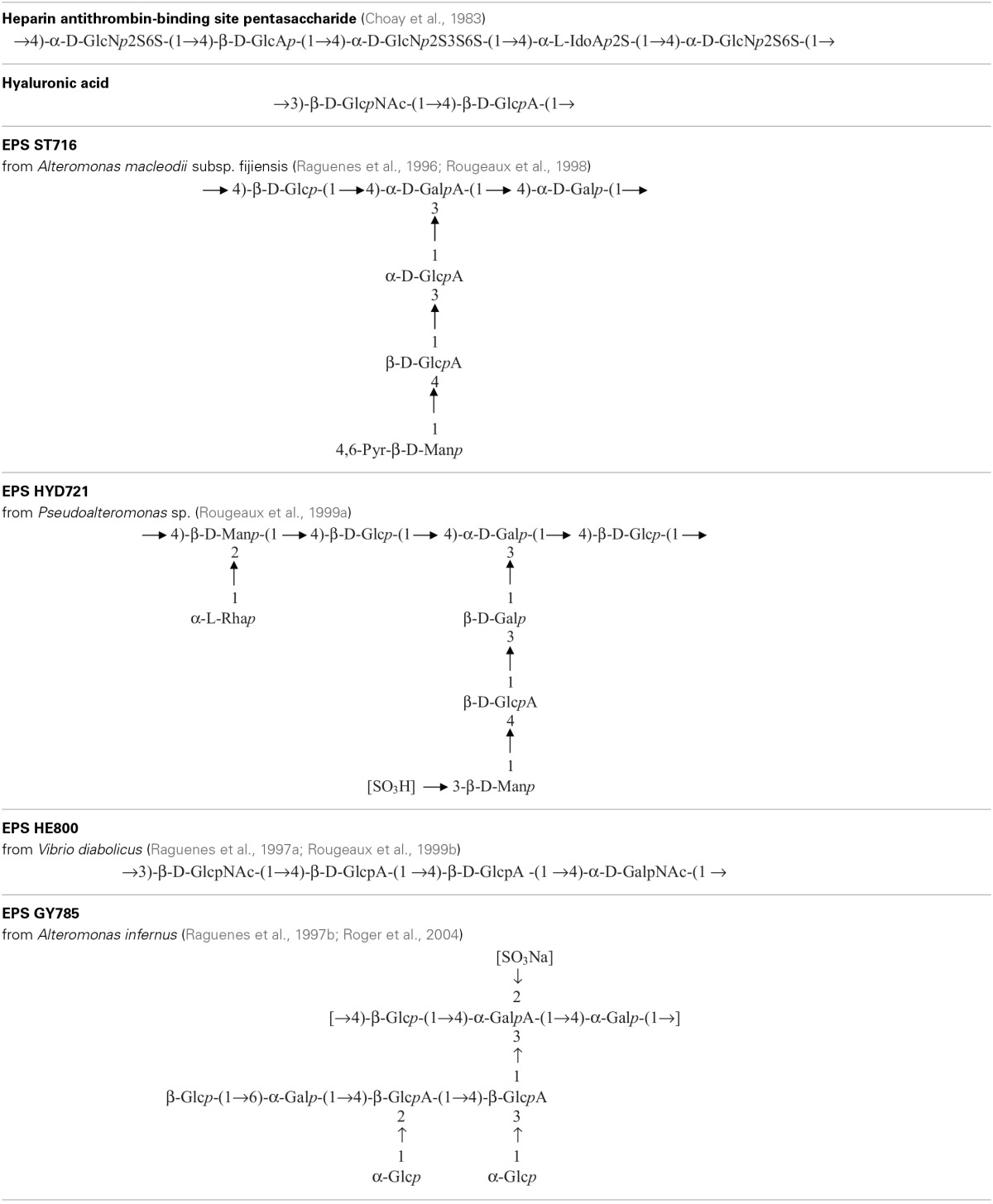
**Repeating unit chemical structures of main glycosaminoglycans and of some marine exopolysaccharides**.

*Galp, galactose; GalpA, galacturonic acid; GalpNAc, N-Acetyl-galactosamine; Glcp, glucose; GlcAp or GlcpA, glucuronic acid; GlcpNAc, N-Acetyl-glucosamine; GlcNp2S6S, N-sulfate-6-O-sulfate-glucosamine; GlcNp2S3S6S, N-sulfate-3,6-di-O-sulfate-glucosamine; GlcNp 2S6S, N-sulfate-6-O-sulfate-D-glucosamine; IdoAp2S, 2-O-sulfate-iduronic acid; Manp, mannose; Pyr, pyruvate group; Rhap, rhamnose; SO_3_H, sulfate group*.

Heparin is a polyanionic molecule of about 80 10^3^ g mol^−1^ with 20 to 40% of sulfates exhibiting several biological activities such as anticoagulant, anti-thrombotic, anti-inflammatory, antiviral and anticancer (Hirsh et al., 2001). It has been discovered in the early twentieth century and used medically for over 70 years. The commercially available drug is extracted from a mucous lining of pig intestines. In parallel, low-molecular weight (LMW) heparin (6–10 10^3^ g mol^−1^) has been developed for use as an anticoagulant to increase benefit-risk ratios (Hirsh et al., 2001). In 1983, the pentasaccharide unit responsible for the anti-coagulant and anti-thrombin activity within the heparin molecule was identified, chemically synthesized (Choay et al., 1983) and it is now commercialized (Petitou et al., 2002) (Table 2). Although this molecule presents, as the unfractionated and LMW heparin, some undesirable bleeding side effects (Hirsh et al., 2001; Crowther and Warkentin, 2008), it remains a lead molecule to study the structure-activity relationship. A heparin contamination crisis arose in the early 2008; some Chinese heparin preparations caused severe side effects resulting in the death of more than 100 patients (Liu et al., 2009). The impurity was identified as a variant of chondroitin sulfate, in which sulfate groups exhibited an unusual pattern (Guerrini et al., 2008). Today, despite the risk of contamination during the extraction process or by animal pathogenic agents, heparin from natural sources continues to be widely used clinically. Still new alternative macromolecules are looked for. In this context, marine microbial polysaccharides stand for a promising source of macromolecules with reduced risk of contamination by mammalian pathogenic agents. New emerging products of high added-value from the marine environment, showing structural homology to heparin such as the presence of sulfate groups, offer the promising opportunity of novel biologically active compounds (Pomin, 2014a).

Exopolysaccharides (EPS) are glycopolymers that microorganisms secrete in their surrounding environment (Sutherland et al., 1972). They can be capsular polymers, which are attached to the cell membrane through the lipopolysaccharides (LPS) anchored in the membrane or other specific proteins (Decho, 1990). EPS can also be produced as a slime loosely bound around the cell or dispersed in the environment (Sutherland, 1982). Bacterial polysaccharides are also present within the membrane cell as a major constituent of the LPS and may be involved in pathogenicity. These glycopolymers have mostly a protective role and permit resistance under extreme environmental conditions by participating in the cell membrane integrity, trapping nutrients, allowing adhesion to surfaces, protecting from toxic compounds and adverse conditions such as freezing (Jannasch and Taylor, 1984; Decho, 1990; Finore et al., 2014). EPS production requires energy from cells representing a carbon investment for microorganisms but the benefits to growth and survival are higher than the production cost (Poli et al., 2011).

## The marine biosphere as a source of new glycopolymers

Representing more than 70% of our planet, ocean is an under-explored and under-exploited vast reservoir for biological organisms and chemical compounds. France owns an expanded maritime domain as well as oceanographic ships, submarine and robots allowing to explore the marine biosphere, and especially deep seas. The marine biosphere is heterogeneous because a large range of ecosystems exist such as microbial mats, Antarctic sea ice, hypersaline marine environments, shallow and deep-sea hydrothermal vents. They are characterized by physical and chemical parameters such as pressure, temperature, pH, chemical compounds, usually toxic. Within the deep-sea hydrothermal vents, large physico-chemical gradients exist; for example, the temperature varies from 2°C, the temperature of the surrounding sea water to the hot temperature of the hydrothermal plume, which can reach 350°C (Baross and Hoffman, 1985). Due to their microbial diversity, these ecosystems might offer promising new biomolecules (Deming, 1998).

### Marine ecosystems with a focus on deep-sea hydrothermal vents

**Figure 1:**
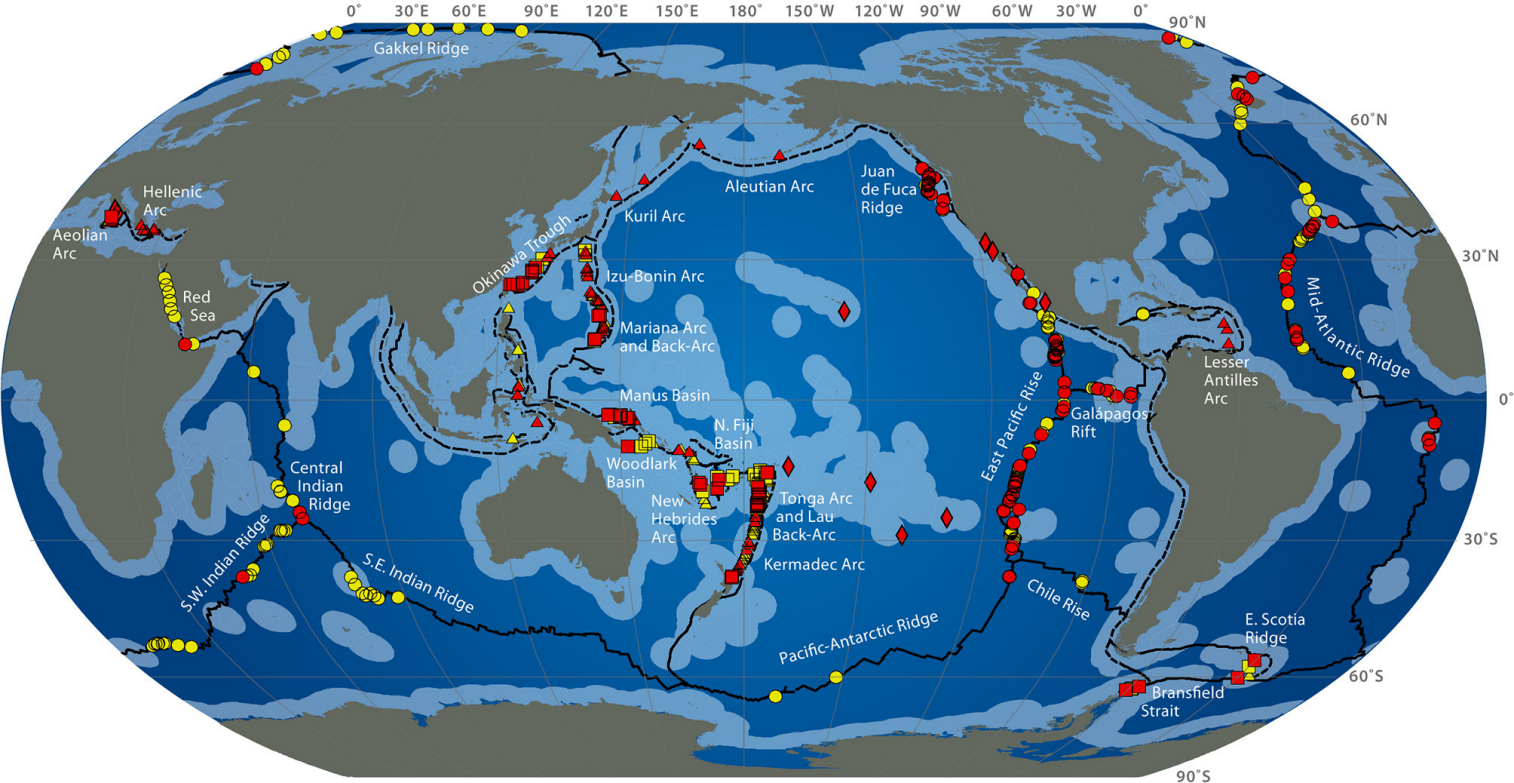
**Global distribution of hydrothermal vent fields (2009) from http://www.interridge.org/irvents/files/Ventmap_2009.jpg, accessed 2014, September 9**. Hydrothermal vent sites from Mid-Ocean Ridge (circles), Arc volcano (triangles), Back-arc spreading center (squares), other (diamonds) are shown (active sites are red, unconfirmed are yellow). Ridges are in plain line, trench are in dashed line. Light blue indicates exclusive economic zones.

Deep-sea hydrothermal vents result from oceanic plate tectonic and submarine volcanic activities. They appear at the sea ridges or on subduction back-arc areas, at a depth of 500 to 4000 m (Figure 1). Seawater at high temperature (up to 350°C) and charged with metals and other compounds such as hydrogen sulfide, hydrogen, ammonia, carbon dioxide flows out of structures built from precipitates called chimneys. Depending on the composition of the fluid, the plume appears with different intensity of white or black color (white or black smokers) (Burgaud et al., 2014). Due to volcanic activities of the crust, these ecosystems are ephemeral (Van Dover et al., 2002). Some other active areas with a diffuse emission of warm or cold water also exist. Deep-sea ecosystems also include cold seeps and sediments or microbial mats (Jannasch and Taylor, 1984). The first hydrothermal vent was discovered in 1977 in the Galapagos area at a depth of 2600 m (Jannasch and Taylor, 1984), although deep-sea biology started with the Challenger expedition (1873–1876). The presence of bacteria in the open ocean seabed is known since the 1880s from the Travaillier and Talisman expeditions (1882–1883) (Zobell and Morita, 1959), when the first microorganisms were found at 5000 m deep (Jannasch and Taylor, 1984). In the 1950s, Galathea expedition collected bacteria-containing sediment samples from 10,000 m deep (Zobell and Morita, 1959).

Deep-sea hydrothermal ecosystems, the most productive ecosystems from the deep ocean, are based on a primary chimiosynthetic production of organic matter sustaining an abundant fauna which is mainly composed of endemic species (Jannasch and Taylor, 1984). These oases of life contrast to the vast surrounding desertic seafloors. A large variety of microbial lifestyles has been described in these ecosystems based on the respiratory type (aerobic-anaerobic), energy and carbon sources. It is adapted to extreme conditions (barophiles needing high pressure, acidophiles-alcalophiles, psychrophiles, mesophiles and extremophiles -growing at temperatures from around 3°C, the temperature of seawater, to 105°C-) (Jannasch and Taylor, 1984). New microorganisms with very diverse metabolisms have then been isolated from marine environment especially in deep-sea habitats (Miroshnichenko and Bonch-Osmolovskaya, 2006). Halophiles are also encountered in some marine ecosystems. The high biodiversity potentially offers a large chimiodiversity. Besides their fundamental interest in basic studies for life origin understanding, chimiosynthetic metabolisms and mechanisms of resistance to high temperatures, these microorganisms arise a great biotechnological interest for the isolation of new enzymes and biomolecules with new or enhanced activities.

### From marine microbial biodiversity to new bioactive molecules

Marine biodiversity is not known currently because only a very small fraction of the microorganisms can be cultivated (Hugenholtz, 2002; Delong et al., 2006). There are reports on particular marine environments with high microbial diversity; this is the case for deep-sea hydrothermal vents in North Atlantic (Sogin et al., 2006). The next generation high throughput DNA sequencing (NGS) methodologies can provide a way to estimate the biodiversity and can be useful to drive some cultivation experiments (Rocha-Martin et al., 2014): when the analysis of metagenomic data sets reveals incomplete pathway for a given compound essential for growth, this compound has to be added as an exogenous source. For example, because SAR11 marine alpha-proteobacteria clade has been shown to be deficient in assimilatory sulfate reduction genes, addition of reduced sulfur compounds in the cultivation medium was tested and was revealed necessary for growth (Tripp et al., 2008).

When the biodiversity increases, the chimiodiversity also increases. Therefore, the chemical study of the marine organisms will most probably result in the discovery of new therapeutic compounds. In particular, it is well recognized that the chemical diversity of marine and microbial compounds is the broadest one (Grabowski et al., 2008). Although microorganisms from the sea have been studied for several decades, their development for biotechnology started only recently (see Imhoff et al., 2011). Today, only a small fraction of the known species has been studied for their biochemical metabolites. Marine microorganisms together with sponges, molluscs, algae, echinoderms are rich source of polysaccharides. Chitin, the second most abundant polysaccharide after cellulose, is found particularly in the shell of crustaceans. Alginates, another marine-derived polysaccharide, are extracted from the cell wall of brown algae (Phaecophyta). Marine-derived polysaccharides naturally bearing sulfate groups are also industrially relevant: carrageenan (red algae), fucoidans (brown algae) and fucans (marine invertebrates) (Berteau and Mulloy, 2003; Kusaykin et al., 2008), ulvan from green seaweeds (Chlorophyta) (Lahaye and Robic, 2007). Microorganisms producing polysaccharides have also been isolated from marine ecosystems especially from deep-sea hydrothermal vents.

Marine chimiodiversity finds applications in human health as therapeutic agents for innovative biological activities. New compounds with new chemical structure can also enable a better understanding of the cellular processes (pathological or not): inhibitors of enzymes involved in inflammatory diseases (manoalide) and phosphorylation processes (okadaic acid), both extracted from sponge, are available for biochemical research (Fenical, 1997). Some examples of marine molecules are already commercialized as analgesic (the peptidic Prialt®), anti-viral (mainly from corals and sponges), anti-cancer (the alkaloid Yondelis® by PharmaMar), or as biomaterials (the calcium carbonate chemical compound Biocoral® by Biocoral from coral). Only a few of them are carbohydrates: alginate of plant origin and chitosan obtained from shrimp extracted chitin have been successfully integrated within wound dressings (Stop Hemo® by Brothier Laboratory in France, Nu-Derm® by Johnson & Johnson in USA) and are also used as encapsulation matrices (geniaBeads® by geniaLab in Germany). Most of the marine molecules are still in research or clinical development, among which four are from marine microorganisms (Imhoff et al., 2011); this leaves the marine biotechnology still at a very promising phase; great efforts are still necessary to get advances in clinical applications.

Bioactive compound prospecting starts with the sampling of organisms. The choices of methods to prepare extracts and to screen for the production of bioactive metabolites are critical steps since they can result in different molecules or even in difficulties to find the activity. The diversification of sources by the exploration of new ecological niches can maintain an effective process leading to innovative results. Compounds should then be isolated, structurally characterized especially including the stereochemistry which can be the main issue for its activity. Biological properties have to be studied in detail, *in vitro* and subsequently *in vivo* before clinical development stages. Alternatively, synthetic analogs can be studied instead of natural molecules. Chemical synthesis would offer access to structurally defined oligosaccharides in sufficient purity and quantity to carry out biological studies. In addition, the synthesis can bring a library of analog compounds slightly differing in the chemical structure as well as in the activity. This library would be a useful tool to establish the structure-activity relationship. Synthetic analogs of the active pentasaccharide in heparin have been well studied due to the high value of this drug and its side effects (Avci et al., 2003). However, synthetic products are sometimes hard to make because of the complexity of the molecule. This is particularly the case for polysaccharides for which only small oligosaccharides have been obtained to date (Boltje et al., 2009). This issue still limits the progress of glycobiology research and much effort are made to develop oligosaccharide synthesis methodologies. Recently, Galan et al. have shown that ionic liquids could be very useful as solvents and promoters of synthetic reactions (Galan et al., 2013).

## Marine exopolysaccharides

### Microbial polysaccharide diversity

Several EPS-producing marine strains have been studied, which led to the discovery and isolation of novel macromolecules (Finore et al., 2014). Marine bacterial polysaccharides have been the subject of several reviews (Guezennec, 2002; Nazarenko et al., 2003; Mancuso Nichols et al., 2005b; Laurienzo, 2010; Satpute et al., 2010; Freitas et al., 2011; Senni et al., 2011; Colliec-Jouault and Delbarre-Ladrat, 2014; Pomin, 2014b). Most of the marine derived EPS are bacterial (classical bacteria i.e., mesophilic and heterotrophic; extremophilic microorganisms i.e., psychrophilic, thermophilic and halophilic); archaea have also been shown to produce EPS (Rinker and Kelly, 2000; Nicolaus et al., 2010; Poli et al., 2011; Finore et al., 2014; Sinquin and Colliec-Jouault, 2014). In the present manuscript, the main focus was put on the extremophilic prokaryotic marine microorganisms that were shown to produce EPS, with an emphasis on bacteria isolated from deep-sea hydrothermal vents.

Mancuso Nichols et al. (2004) described the production of EPS by the marine strains *Pseudoalteromonas* CAM025 and CAM036 isolated in Antarctica sea water and sea ice. Some other strains from Arctic sea ice have been shown to produce EPS with cryoprotective effect (Liu et al., 2013).

The halophilic strains *Halomonas maura* (Arias et al., 2003), *Halomonas ventosae* (Martinez-Canovas et al., 2004), *Halomonas alkaliantarctica* (Poli et al., 2007), *Hahella chejuensis* (Poli et al., 2010) and the archaeal halophilic *Haloferax mediterranei* (Anton et al., 1988; Parolis et al., 1996) isolated from hypersaline environments were also shown to produce EPS; some of them are sulfated (Poli et al., 2010).

Marine thermophilic anaerobes *Sulfolobus, Thermococcus* and *Thermotoga* were described to produce EPS (Vanfossen et al., 2008). *Thermococcus litoralis* produces an EPS which contains sulfate and phosphorus substituents (Rinker and Kelly, 2000); the archaea *Sulfolobus solfataricus* has also been shown to produce a sulfated polysaccharide (Nicolaus et al., 1993). *Geobacillus* sp., *Bacillus thermodenitrificans* and *B*. *licheniformis* thermophilic strains have been isolated from shallow marine hydrothermal vents of Vulcano Island (Italy) and the polysaccharide they produce has been analyzed (Poli et al., 2010).

Several bacteria belonging to gamma-proteobacteria Alteromonadales or Vibrionales orders and isolated from the deep-sea polychaete annelids *Alvinella pompejana* and *A. caudata* tissues have been shown to produce different EPS: HYD1545 (Vincent et al., 1994), HYD1644 (Dubreucq et al., 1996), HYD721 (Rougeaux et al., 1999a), HYD657 produced by *A. macleodii* subsp. *fijiensis* biovar deepsane (Cambon-Bonavita et al., 2002) and HE800 produced by *Vibrio diabolicus* (Raguenes et al., 1997a; Rougeaux et al., 1999b). This latter polysaccharide possesses a particular hyaluronic acid-like chemical structure and contains both hexosamines and uronic acids alternating in the repeating unit sequence (Table 2) (Sinquin and Colliec-Jouault, 2014).

Another gamma-proteobacterium isolated from a deep-sea hydrothermal vent shrimp (*A. macleodii* subsp. *fijiensis* biovar *medioatlantica*) was shown to produce the EPS MS907 (Raguenes et al., 2003) whereas *Alteromonas macleodii* subsp. *fijiensis* producing the pyruvated EPS ST716 (Raguenes et al., 1996; Rougeaux et al., 1998) and *Alteromonas infernus* producing the sulfated EPS GY785 were isolated from hydrothermal fluids (Raguenes et al., 1997b; Roger et al., 2004) (Table 2).

Other *Pseudoalteromonas* strains produce diverse EPS: SM9913 isolated from deep-sea sediments produces an acetylated EPS (Qin et al., 2007), SM20310 from the arctic sea ice produces a complex α-mannan (Liu et al., 2013).

*Pseudomonas, Alteromonas, Paracoccus* and *Vibrio* sp. bacteria producing EPS under laboratory conditions have also been isolated from microbial mats in French Polynesia, another marine ecosystem considered as extreme. Among them, *Paracoccus zeaxanthinificiens* subsp. *Payriae* and *Vibrio* sp. RA 29 are described to produce sulfated polysaccharides and *Vibrio* sp. MO 245 would produce a polymer very similar to the *Vibrio diabolicus* one (Guézennec et al., 2011).

When known, the chemical and structural diversity of these few molecules confirms the high value of the marine environment as a source of exciting chimiodiversity.

### Alginate and chitosan, two major marine polysaccharides

Alginate traditionally extracted from brown algae and the most important polysaccharide from this type of seaweeds is also produced by soil bacteria *Azotobacter* and *Pseudomonas* species as an exopolysaccharide (Gomez D'ayala et al., 2008). Although they are not marine bacteria, the polysaccharide is usually refered as marine derived. A gene cluster for alginate biosynthesis have been identified in *Vibrio* sp. QY102 suggesting this polymer would be involved in the formation of biofilm by *Vibrio* sp. QY102 (Shi et al., 2008).

The bacterial alginates are all composed of mannuronic (M) and guluronic (G) acids arranged in homo-blocks (M or G) and hetero-blocks (MG) as algal alginates; but that from *Pseudomonas* does not have block G; in addition many of bacterial alginates are acetylated. G-rich alginates and M-rich alginates have different physico-chemical and biological properties (Colliec-Jouault and Delbarre-Ladrat, 2014). Although their osidic composition is similar, bacterial alginates vary considerably in their structure depending on the producing strain and in the case of alginate produced by seaweeds, on seasonal and environment variations (Gomez D'ayala et al., 2008; Bertagnolli et al., 2014). Industrial production of alginate reaches 30,000 tons annually and it is used as a viscosifier, stabilizer and gelling agent (Hay et al., 2013).

Chitin and chitosan, its partially N-deacetylated derivative, are the most abundant marine polysaccharides; they have great interest for biotechnological applications. They are mainly extracted from shellfish waste. To date no bacterium has been described to produce either chitin or chitosan. Nonetheless, some bacteria have been shown to produce PNAG, poly-*N*-acetyl-glucosamine, a β-1,6-linked *N*-acetyl-D-glucosamine homopolymer, structurally close to chitin, β-1,4-linked *N*-acetyl-D-glucosamine homopolymer (Whitney and Howell, 2013; Ye et al., 2014).

### Production and physiological conditions influence

Production of polysaccharides by bacteria is a physiological process that takes place under a diversity of environmental conditions (Kumar et al., 2007), usually in response to a stress including the adaptation to extreme environment (Lapaglia and Hartzell, 1997). In the marine environment, bacteria are usually attached to biotic or abiotic surfaces and are embedded in a slime, an exopolymeric matrix composed of proteins, polysaccharides, DNA and small organic molecules; they form biofilms as a protective response to a stress (Lapaglia and Hartzell, 1997; Guezennec, 2002; Liu et al., 2013). Therefore, it is important to identify any relationship between the biosynthesis of EPS and biofilm formation regulation in order to better control the production process (Rehm, 2010). Limitations in the availability of nutrients such as nitrogen, phosphorous, sulfur, potassium (Kumar et al., 2007; Sabra and Hassan, 2008), as well as temperature, osmotic or pH shifts (Gorret et al., 2001; Kumar et al., 2007) are stressing factors enhancing EPS production. Moreover, a suitable carbon source to be added to the production or growth medium is also a main factor for the yield and features of the produced molecule (Donot et al., 2012). Its excess, in fact, is concomitant with another component limitation such as nitrogen (Freitas et al., 2011). In some cases, the osidic composition of the molecule can change with the carbon substrate (Grobben et al., 1996; Poli et al., 2007; Freitas et al., 2011).

Production yield of marine EPS is usually around 1 g L^−1^ (Decho, 1990). Industrial development is conceivable for a production yield around 10 g L^−1^. Production costs are driven by the yield of polysaccharide, the amount and cost of carbon source, and by the downstream processes needed for molecule separation. Therefore, studies on production optimization are needed even if some bacterial strains are naturally able to produce EPS at a high yield (e.g., 50 g L^−1^ or over) such as *Agrobacterium* sp. (curdlan), *Xanthomonas campestris* (xanthan), *Zymomonas mobilis* (levan), *Alcaligenes faecalis* (curdlan)*, Bacillus* sp. (levan) (Donot et al., 2012). Factors such as medium composition and fermentation conditions i.e., temperature, pH and aeration stand for the main studied variables to optimize the fermentation process (Mancuso Nichols et al., 2005a; Sabra and Hassan, 2008; Finore et al., 2014). The nutritional conditions can also affect the molecular weight as well as the osidic composition of the EPS in some cases (Mancuso Nichols et al., 2005a; Poli et al., 2009; Donot et al., 2012; Finore et al., 2014). A novel *Alteromonas macleodii* strain has been shown to produce 23.4 g L^−1^ exopolysaccharide when grown on 15% lactose which is the highest yield obtained for marine EPS (Mehta et al., 2014).

Fermentation is usually performed in batch or fed-batch. Heat transfer and oxygen supply limitations are however encountered when EPS are highly viscous (Kreyenschulte et al., 2014). Depending on the producing bacterium, the EPS may be produced during the growth (cellulose, gellan or alginate by *Azotobacter vinelandii*), when the growth has ended (curdlan by *Alcaligenes faecalis*), during the stationary phase (Raguenes et al., 1996) or both (xanthan by *X. campestris*) (Decho, 1990; Freitas et al., 2011). The marine EPS HYD1644 is produced only after the initial exponential growth phase (Samain et al., 1997). In *Pseudomonas* sp., an EPS is produced during the exponential phase and another one with a different chemical structure is produced during the stationary phase (Christensen et al., 1985). Some reports emphasize the benefit of a growth-production uncoupling by using a bacteriostatic to stop the growth together with the addition of the carbonated substrate and/or shift in temperature or pH (Looijesteijn and Hugenholtz, 1999).

### Downstream process for EPS recovery

After fermentation, the downtream process for the recovery of EPS is an important step for the production costs and commercial value of the molecule. Process steps will depend on the molecule, on other undesired molecules produced, but also on the level of purity required.

Downstream process, consisting of the removal of insoluble particles, isolation of the product and further purification, is based on classical steps of extraction and purification. Each method has its advantages and disadvantages (Sinquin and Colliec-Jouault, 2014). Various molecules are usually associated with polysaccharides that act as sponges for some proteins, amino acids, DNA, RNA, salts, metals, fatty acids either dissolved in seawater based medium or released after cell lysis (Decho, 1990; Freitas et al., 2011; Donot et al., 2012). The presence of degrading enzymes or of a second biopolymer must also be taken into account for a good purification process (Kreyenschulte et al., 2014). Therefore some isolation procedures may be better adapted than others as reviewed by Donot et al. (2012).

Centrifugation or filtration step is usually used to remove cells from the culture broth without lysis and EPS isolation (Kreyenschulte et al., 2014). Culture broth is sometimes subjected to heating at the end of the fermentation to kill the bacteria and denature potential polymer-degrading enzymes (Freitas et al., 2011) but this may result in some cell lysis and release of compounds in the soluble medium. The deactivation of the cells is alternatively carried out by chemical, enzymatic or mechanical treatment (Kreyenschulte et al., 2014).

Separation and purification of the biopolymer can be obtained by precipitation with the addition of alcohol or by complexing metal ions (Kreyenschulte et al., 2014). However, ethanol precipitation may trap and co-precipitate proteins and ions (Kumar et al., 2007) and needs a large amount of alcohol. Filtration or ultrafiltration is recognized as a good method to separate high molecular weight from other small adsorbed compounds and has been used since a long time (Wilkie et al., 1957; Kreyenschulte et al., 2014). Some additional extractions to remove contaminating compounds may be appropriate such as new precipitation, chemical extraction or enzymatic treatment but they may decrease the recovery yield (Decho, 1990; Freitas et al., 2011). These separation steps can in turn be hindered when polysaccharide is highly viscous; higher temperature or dilution in water may facilitate the process however increasing the costs (Freitas et al., 2011). The choice of the whole procedure has to be adapted to polymer characteristics as well as to the desired recovery yield, purity and integrity degrees. After isolation, the polysaccharide is freeze dried for a better conservation. Polysaccharides are highly hydrophilic due to hydroxyl groups, especially when they are polyanionic (carboxyl groups), a widespread feature in marine environment, or when they bear sulfate groups; therefore they always conserve a content of water (De Angelis et al., 2013).

### Putative pathways of biosynthesis

The genetics of the EPS biosynthesis begins to be better understood; however, information appears disparate because it depends on the concerned microorganism and the polysaccharide that it produces.

However, depending on the type of polysaccharide, some general mechanisms can be described (Rehm, 2010). An extracellular glycosyltransferase (GT) is responsible for the biosynthesis outside the cell of homopolysaccharides such as dextran, levan, mutan. This particular GT (usually sucrase) cleaves a disaccharide substrate (usually sucrose) and transfers one of the two obtained monomers to the polymer chain (Rehm, 2010). The occurrence of these enzymes is however limited in marine microorganisms (Decho, 1990).

Except for mechanisms involving sucrase, the biosynthesis starts with the production of nucleotide sugars which will be linked in the repeating unit of the molecule. They are biosynthesized within the central cellular metabolism with usually known enzymes (Rehm, 2010). The repeating unit is then synthesized by appropriate GTs (Whitfield, 2006; Rehm, 2010). After completion, the repeating unit is exported outside the cell and polymerized on the growing EPS chain. Based on biosynthesis and export mechanisms, three pathways have been described.

Some simple heteropolysaccharides (two different residue types maximum such as hyaluronic acid) as well as some homopolysaccharides (cellulose, chitin) are synthesized by a synthase enzyme which polymerizes nucleotide sugars while exporting the growing polymer chain outside the cell (Weigel and De Angelis, 2007; Rehm, 2010). The second pathway relies on the ABC transporter for exportation of the entire polysaccharide synthesized on a lipid carrier (Whitney and Howell, 2013). The third mechanism depends on Wzx-Wzy proteins.

The Wzx-Wzy dependent mechanism has been widely studied in Gram negative bacteria especially for heteropolysaccharide production. Biosynthesis is catalyzed by a membrane-spanning multiprotein complex (Rehm, 2010). A particular GT involved in the initiation step, the phosphoglycosyltransferase (pGT), anchors the first osidic residue to a membrane lipid carrier (undecaprenyl phosphate) through a phosphoryl bond (De Vuyst et al., 2001; Whitfield, 2006). After completion of the repeating unit by successive GTs, it is exported outside the cell across the inner membrane by Wzx and subsequently polymerized by the Wzy protein (Whitfield, 2006). The lipid carrier anchor is also recognized for the translocation of the repeating unit across the inner membrane (Rehm, 2010). The final translocation across the outer membrane involves a member of the outer membrane polysaccharide export protein family such as Wza (Reid and Whitfield, 2005). Heteropolysaccharides from the Gram-positive lactic acid bacteria are synthesized by a very similar mechanism (De Vuyst et al., 2001).

Besides these four types of biosynthesis mechanisms (extracellular, synthase-, Wzx-Wzy- and ABC-transporter), some other gene clusters showing a peculiar mechanism have been described. In general, the overall regulation of the biosynthesis is not completely understood as well.

Sphingans are heteropolysaccharides produced by *Sphingomonas* bacteria, some of whom are of marine origin (Cavicchioli et al., 1999). These polymers are characterized by a tetrasaccharide backbone structure containing rhamnose or mannose (1), glucose (2) and glucuronic acid (1). Gellan, diutan, welan, rhamsan, and sphingan S-88 are examples of them differing by their side chain and substituents (Harding et al., 2004; Freitas et al., 2011). The biosynthesis of sphingans has been recently reviewed by Schmid et al. (2014). The organization of genes required for diutan, welan, gellan, S-88 and S-7 biosynthesis shows similarities; genes for polysaccharide and protein secretion, as well as an operon for the synthesis of dTDP-rhamnose are conserved suggesting a well-conserved mechanism for polysaccharide biosynthesis and secretion (Yamazaki et al., 1996; Harding et al., 2004; Coleman et al., 2008). The mechanism starts by the transfer of glucose-1-phosphate on the isoprenylphosphate lipid. Successive GTs transfer the other sugar nucleotide to the repeating unit similarly to the first steps of Wzx-Wzy dependent mechanisms (Coleman et al., 2008; Schmid et al., 2014). A putative gene for lyase is only present in diutan gene cluster. Comparison of the three gene clusters allowed also the identification of a candidate gene encoding the protein responsible for the addition of rhamnosyl side chain.

Alginate biosynthesis genes in *Azotobacter* and *Pseudomonas* bacteria are similar (Rehm and Valla, 1997), the 12-core genes are clustered in a single operon. Only slight differences exist especially in the regulation (Donot et al., 2012; Hay et al., 2013). Alginate is synthesized as polymannuronnic acid in an undecaprenol-independent manner (Remminghorst and Rehm, 2006) and is *O*-acetylated in the periplasm. The gene cluster includes genes for the precursor synthesis (GDP-mannuronic acid), the polymerization, the translocation across the inner membrane and the periplasm where alginate encounters some modifications (*O*-acetylation and C5-epimerization of mannuronic acid to guluronic acid), and the alginate secretion (Remminghorst and Rehm, 2006; Hay et al., 2013). In the case of alginate biosynthesis by *Azotobacter vinelandii* and *Pseudomonas fluorescens*, the molecular mechanisms of polymerization and export are not fully understood (Rehm, 2010), but are based on a synthase-dependent mechanism (Whitney and Howell, 2013).

The groups of genes necessary for the biosynthesis of an heteropolysaccharide are typically clustered at one genetic locus of 12–25 kb including genes for the synthesis of the repeating unit (GTs), genes for export, polymerization and regulation (Laws et al., 2001). These clusters are also well known in lactic acid bacteria (LAB) such as *Streptococcus* spp. (Wu et al., 2014) and *Bifidobacterium* spp. (Hidalgo-Cantabrana et al., 2014); in LAB, EPS biosynthesis gene clusters are often located on plasmids (Kumar et al., 2007; Donot et al., 2012). Furthermore, *Escherichia coli* polysaccharide biosynthesis pathways have become a reference model in these studies (Whitfield, 1995, 2006; Willis and Whitfield, 2013).

The activated precursors are also needed for the synthesis of some other cellular components such as peptidoglycan for membranes (Merritt et al., 2013). Therefore, it is of high importance to understand the fluxes of carbon, nitrogen and energy leading both to cells and bioactive molecules or, at least, to modulate them by physiological optimizations or genetic engineering (Rehm, 2010). It is highly probable, due to their respective role, that cell wall biosynthesis has priority, LPS and finally EPS synthesis (Decho, 1990).

With the post-genomic era, more clusters involved in biosynthesis of polysaccharides are described together with some regulations issues. An understanding of how the high-molecular weight polymers are biosynthesized may lead to better efficiencies in EPS production at an industrial level and a better comprehension of how varies the composition upon production conditions. New strategies like genetic engineering of producing microorganisms are being developed to enhance polysaccharide yield and allow an economically effective production (Ates et al., 2011; Finore et al., 2014). On the other hand, the prediction of metabolic network can allow the identification of key factors for an enhanced production: from the genome sequence, mannitol was identified as a stimulator for levan biosynthesis by *Chromohalobacter salexigens* (Ates et al., 2011), experimental evidence was achieved later in another halophilic bacterium *Halomonas smyrnensis* (Ates et al., 2013). Metabolic engineering approaches can complement ongoing efforts on fermentation process engineering with the aim to optimize EPS production (Merritt et al., 2013). In this strategy, research on K5 EPS biosynthesis is a good example as reviewed by Wang et al. (2011). Fermentation process has been optimized for a better yield and productivity of heparosan. Conversion of UDP-glucose to UDP-glucuronic acid by the UDP-glucose dehydrogenase together with the UDP-*N*-Acetyl-glucosamine pathway have been identified as limiting steps to keep balanced supply of nucleotide sugars both for heparosan biosynthesis and cell wall synthesis. Genetic engineering targeting these metabolic reactions has revealed the necessity of a balanced over-expression of KfiA and KfiC GTs. Since a part of the K5 polysaccharide remains linked to the cell membrane, the gene of the K5 lyase capable of breaking this linkage has been genetically modified to increase the amount of K5 released in the supernatant (Wang et al., 2011). K5 lyase gene can also be genetically engineered to control the chain length (Wang et al., 2011).

### Novel strategies for chemical and enzymatic modifications

Overall, physical and bioactive features of polysaccharides are based on molecular chemical structure: the osidic sequence and linkages in the repeating unit, but also the substituents, influence the conformation and geometry of polysaccharide chains as well as polysaccharide-polysaccharide and polysaccharide-protein interactions (Powell et al., 2004; Pomin, 2009). The chemical structure determines the physical properties such as solubility in water or interactions with ions (Geddie and Sutherland, 1993; Kumar et al., 2007). For examples, the acetyl content in chitosan sulfate influences the inhibition of propyl endopeptidase which is involved in progressive memory deficits and cognitive dysfunctions (Je et al., 2005). The sulfate groups in heparin participate in the molecular conformation and influence the binding with calcium cations (Chevalier et al., 2004). They also have a great effect on the anticoagulant activity (Franz and Alban, 1995; Garg et al., 2002; Huang et al., 2003; Liu and Pedersen, 2007). The antiproliferative activity of the heparin molecule depends on the molecular size but not on 3-*O*-sulfo group (Garg et al., 2002) and requires both *N*-acetylation and *N*-sulfation (Longas et al., 2003). As found for heparin structure-function relationships, the amount of sulfate groups, their distribution pattern and the molecular weight are of great importance for GAG-like activities. Therefore, marine polysaccharides may be structurally modified e.g., depolymerized and (over-)sulfated to render them active or to enhance already existing activities (Chopin et al., 2014).

Chemical modifications are widely used for this purpose (Gomez D'ayala et al., 2008; Laurienzo, 2010; Senni et al., 2011): acid hydrolysis (Colliec et al., 1994), radical depolymerization (Nardella et al., 1996), *N*-deacetylation (Zou et al., 1998), sulfation (Nishino and Nagumo, 1992; Guezennec et al., 1998). However, several drawbacks such as lack of control and regioselectivity, use of organic solvents, non-homogeneous conditions are identified (Al-Horani and Desai, 2010). More recently, ionic liquids have been used for cellulose sulfation in homogeneous media (El Seoud et al., 2007; Gericke et al., 2011). Only few reports deal with chemical modifications on marine EPS (Gomez D'ayala et al., 2008). Low-molecular weight oversulfated derivatives of the EPS GY785 from the deep-sea bacterium *A. infernus* and of the EPS HE800 from *V. diabolicus* have been obtained by depolymerization by acid hydrolysis or free-radical reaction followed by sulfation with sulfur trioxide pyridine complex (Colliec-Jouault et al., 2001; Senni et al., 2011). The low-molecular weight and oversulfated derivatives thus obtained exhibit biological activities similar to heparin and other GAGs (Ruiz Velasco et al., 2011; Senni et al., 2011, 2013; Sinquin and Colliec-Jouault, 2014).

Target compound yield by chemical process is low after purification steps and sulfation lacks specificity giving undesirable by-products or uncontrolled final product chemical structure resulting in non-homogeneous products (Chopin et al., 2014). Enzymes, because of their specificity, may allow a better control of the reactions catalyzed. Moreover, enzymatic reactions are more friendly to the environment without the need of solvent or toxic chemicals.

To date, several strategies combining chemical and enzymatic methods for the synthesis of GAGs have been developed (De Angelis et al., 2013). After sugar backbone isolation or synthesis, modifications, usually using enzymes cloned from vertebrates up to now, are performed *in vitro*.

*Escherichia coli* K5 polysaccharide has the same chemical structure as the biosynthetic precursor of heparin (heparosan); it has been modified by combinations of chemical and enzymatic methods and converted to “biotechnological heparin” (Kusche et al., 1991; Naggi et al., 2001; Urbinati et al., 2004; Lindahl et al., 2005). *N*-deacetylation and *N*-sulfation were achieved by chemical reactions, and C5-epimerization of glucuronic acid (GlcA) to iduronic acid (IdoA) was carried out using the enzyme epimerase. The sulfation reaction was not regioselective enough and was improved in 2005 to yield the desired 2-*O*-sulfated IdoA (Lindahl et al., 2005); at that time, even if the chemical structure of this neoheparin was still not identical to the heparin AT-binding sequence, biological properties were similar to those of heparin. Other similar chemoenzymatic processes have been patented (Zopetti et al., 2006).

The sulfation step is crucial in these biotechnological processes. Although chemical sulfation is not specific enough, only a few biotechnological processes involve sulfotransferases (STs). In particular, a selectivity toward the sulfation state of the substrate exists (De Angelis et al., 2013). Enzymatic sulfations are however limited by the need of the expensive sulfate donor 3′-phosphoadenosine 5′-phosphosulfate (PAPS). Chondroitin sulfate E has been synthesized from chondroitin sulfate A using a sulfotransferase extracted from squid cartilage (Habuchi et al., 2002). In this study, dermatan sulfate has also been oversulfated with the same enzyme. Enzymatic sulfation has been used to prepare heparan sulfate from chemically desulfated *N*-sulfated heparin (Chen et al., 2005). Using heparan sulfotransferases (2-OST, 6-OST, 3-OST) expressed in *E. coli* and immobilized, as well as PAPS regeneration system, the polysaccharide substrate was subjected to different enzymatic modifications resulting in heparan sulfates with distinct biological activities.

However, although promising, chemoenzymatic methods still need more research efforts to allow the synthesis of GAG-like macromolecules. These multiple step processes are expensive due to the PAPS cost and the production capacity remains limited for a demand in heparin of about 100 tons per year (De Angelis, 2012).

Chitin and chitosan have been extensively studied for their biotechnological applications and have been subjected to a large range of modifications to modulate their biological properties, hence determining their applications (Gomez D'ayala et al., 2008). Removal of acetyl groups (deacetylation) from chitin is the first of the studied modifications among others including carboxymethylation, sulfation, acylation (Gomez D'ayala et al., 2008).

A new alternative to chemical and enzymatic modifications to obtain targeted polysaccharide chemical structure could also be the genetic engineering. This will be feasible if more EPS structures are known together with biosynthetic genetic clusters as well as relationship between structure and bioactivity. EPS biosynthesis pathway reconstruction is feasible with genome sequence and functional annotation and it can be engineered to obtain tailor-made polymers (Schmid et al., 2014). If biosynthesis mechanisms are known, the over-expression or inhibition of targeted genes may lead to enhanced EPS production. It may also allow a better insight into protein function as well as the biosynthesis of tailored molecules with desired features in terms of molecular weight, osidic structure and functional substituents. The activity of GT GumK involved into the biosynthesis of xanthan has been modified by protein engineering; this resulted in a variation in the xanthan production yield highlighting the possibility to obtain tailor-made xanthan molecules by GT engineering (Barreras et al., 2008). Attempts to determine genes involved in the molecular weight control of diutan in *Sphingomonas* sp. were not successful (Coleman et al., 2008), suggesting that the chain length regulation is very complex whatever the biosynthesis mechanism considered (Whitfield, 2010). In *Pseudomonas aeruginosa*, the GDP-mannose dehydrogenase has been identified by over-expression studies as a key regulator protein in alginate biosynthesis (Tatnell et al., 1994).

On the other hand, genomic data would also allow the identification of new enzymatic tools to modify glycopolymers. Enzymes would become promising biotechnological tools for *in vitro* synthesis or *in vivo* biosynthesis engineering. The marine biodiversity has already shown a great potential in providing new biocatalysts (Trincone, 2010). Depolymerising enzymes can be used in *in vitro* depolymerization process but also as tools to study the chemical structure. Carbohydrate sulfotransferases and other enzymes grafting substituents such as acetate, as well as enzymes catalyzing their removal, may be used *in vitro* for binding or for elimination of substituents which are of great importance for the final bioactivity of the molecule. GTs specificity are difficult to characterize especially when they belong to the polyspecific family 4 or 2 (http://www.cazy.org/). Identifying both the polysaccharide chemical structure and the genetic cluster for the biosynthesis would allow the knowledge of the GT enzymatic function in the biosynthetic pathway by genetic knockout. It would also provide useful directions to determine enzymatic specificity and characterize the enzymes.

Recombinant production can be envisioned when polysaccharide biosynthesis cluster is known: it consists in the cloning of the entire biosynthesis cluster in an appropriate heterologous host. This is a new strategy developed together with the expansion of synthetic biology (Winter and Tang, 2012; Cameron et al., 2014; Church et al., 2014). Up to now, only less complex polymers have been studied in recombinant production such as hyaluronic acid (Chien and Lee, 2007; Cimini et al., 2011), chondroitin (Ninomiya et al., 2002) and heparosan (Roman et al., 2003).

## Concluding remarks

Natural bioactive molecules attract many interests in the search for new therapeutic drugs. Marine environment shields a high diversity of natural products and can be indeed a treasure chest for industrial and pharmaceutical purposes. Due to their functions in survival and competitiveness of marine bacteria in low nutrients and adverse environments, the EPS are ubiquitous in the marine environment.

In addition to various marine origins (animals, seaweeds, invertebrates), microorganisms provide glycopolymers with original chemical structure and promising biological activities. The marine biotechnology has not yet reached an economically significant field but is a promising field for sustaining convenient macromolecules.

It is also assumed that new isolated bioactive molecules are also the matter for bioinspired new molecules obtained by synthesis. For this purpose, both the discovery of new natural bioactive molecules and their structural characterization are still of considerable importance to obtain well characterized molecules with truly identified mechanism of action. Basic and applied research efforts in this field require a close collaboration between biologists and chemists and expertise in marine microbiology, biochemistry, biology, chemistry and computational sciences to fulfill screenings, structural characterization, bioactivity studies and biosynthesis understanding and metabolic engineering issues. With the synthetic biology approach, a new era is available for the polysaccharide production field by simple ways.

### Conflict of interest statement

The authors declare that the research was conducted in the absence of any commercial or financial relationships that could be construed as a potential conflict of interest.
